# Metabolic, Enzymatic Activity, and Transcriptomic Analysis Reveals the Mechanism Underlying the Lack of Characteristic Floral Scent in Apricot Mei Varieties

**DOI:** 10.3389/fpls.2020.574982

**Published:** 2020-10-22

**Authors:** Fei Bao, Tengxun Zhang, Anqi Ding, Aiqin Ding, Weiru Yang, Jia Wang, Tangren Cheng, Qixiang Zhang

**Affiliations:** ^1^Beijing Advanced Innovation Center for Tree Breeding by Molecular Design, Beijing Forestry University, Beijing, China; ^2^Beijing Key Laboratory of Ornamental Plants Germplasm Innovation & Molecular Breeding, National Engineering Research Center for Floriculture, Beijing Laboratory of Urban and Rural Ecological Environment, Key Laboratory of Genetics and Breeding in Forest Trees and Ornamental Plants of Ministry of Education, School of Landscape Architecture, Beijing Forestry University, Beijing, China

**Keywords:** floral scent, benzyl acetate, eugenol, benzyl alcohol, benzyl benzoate, PmBAR, apricot mei, *Prunus mume*

## Abstract

Apricot mei, a hybrid of *Prunus mume* and *Prunus sibirica*, usually has greater cold resistance than *P. mume*; however, most varieties of Apricot mei lack the characteristic floral scent of *P. mume*. The volatile and intracellular metabolites, activity levels of key enzymes, and transcriptomes of blooming flowers were comprehensively investigated in five varieties of *P. mume*. Benzyl acetate and eugenol were determined to be the main components of the *P. mume* floral scent. However, benzyl benzoate and benzyl alcohol benzoyltransferase activity was detected in only the low-fragrance varieties “Dan Fenghou” and “Yanxing.” No benzyl alcohol or benzaldehyde reductase (BAR) activity was detected in the non-fragrant variety “Fenghou.” *PmBAR1* and *PmBAR3* were identified as the key genes responsible for BAR activity. The lack of benzyl alcohol synthesis in the “Fenghou” variety was caused by low activity of PmBAR1-Fen and low expression of *PmBAR3*. The 60-aa segment at the N-terminus of PmBAR3 was found to play an important role in its enzymatic activity. Correlation tests between floral scent metabolites and the transcriptomes of the five different scented varieties showed that some transcripts associated with hormones, stresses, posttranslational modifications and transporters may also play important regulatory roles in floral scent metabolism in the different varieties.

## Highlights

- Activation of the benzyl benzoate pathway and inhibition of the benzyl alcohol synthesis pathway are the two main reasons for defects in floral scent in varieties of apricot mei.

## Introduction

*Prunus mume* (mei) is a traditional flower in China with a long cultivation history; it produces aromatic flowers in the early spring. Mei originated in the southwestern part of China around the Yangtze River and was later introduced to North China. Due to the poor cold resistance of *P. mume*, few varieties can grow in the open fields of North China, except for some hybrids of species of different genera. Both *P. mume* and *Prunus sibirica* are diploid (2n = 16). The inbred varieties of *P. mume* and *P. sibirica* can survive in small numbers, and natural hybrids of them are found. The cold tolerance of *P. sibirica* is greater than that of *P. mume*. The hybrids between *P. mume* and *P. sibirica* have been named apricot mei, which have greater cold resistance than most *P. mume* varieties and are suitable for cultivation in North China. However, most varieties of apricot mei lose the characteristic floral scent of *P. mume*.

The main components of the characteristic floral scent of *P. mume* are benzyl acetate and eugenol (Hao et al., 2014a, b). The synthesis pathways of benzyl acetate and eugenol both belong to the phenylpropene metabolism pathway, which has been reported and studied more clearly in the model plants petunia and *Arabidopsis* (Fraser and Chapple, 2011; Dudareva et al., 2013; Muhlemann et al., 2014; Widhalm and Dudareva, 2015). PAL, which can catalytically deaminate L-phenylalanine, is responsible for the initial step of phenylpropanoid metabolism (Cochrane et al., 2004). Benzaldehyde is produced in two ways, including CoA-dependent, β-oxidative and CoA-independent non-β-oxidative pathways in petunia (Boatright et al., 2004). The acyltransferases play important roles in phenylpropene metabolism. Synthesis of benzyl acetate is catalyzed by BEAT using benzyl alcohol and acetyl-CoA as substrates. BEAT belongs to the BAHD acetyltransferase family, which is a type of acyl-CoA dependent acetyltransferase). Benzyl benzoate is produced by BPBT using benzyl alcohol and benzoyl-CoA as substrates (Boatright et al., 2004). Benzyl alcohol is reduced from benzaldehyde. The specific enzyme xylB, which reduces benzaldehyde to benzyl alcohol, has been identified in *Pseudomonas* (Suhara et al., 1969), but it has not been clearly identified in plants. However, rose PAR was reported to catalyze the reduction of benzaldehyde to benzyl alcohol with half activity (Chen et al., 2011). The expansion of *PmBEAT* genes in the genome produced some genes that were highly expressed in the flowers, which resulted in the unique species characteristics in floral scent of *P. mume* (Zhang et al., 2012; Bao et al., 2019).

Eugenol is another main component of the characteristic floral scent of *P. mume*. The production of eugenol in petunia is catalyzed by EGS1 using coniferyl acetate and NADPH as substrates (Koeduka et al., 2006). Coniferyl acetate is synthesized from coniferyl alcohol by CFAT, which also belongs to the BAHD acetyltransferase family. The functional *PmCFAT* gene in *P. mume* has been identified and characterized (Zhang et al., 2019b). Since coniferyl alcohol is one of the precursors of monolignol, 4CL, CCR, CAD, and HCT, which affect lignin synthesis, they can also catalyze the synthesis of eugenol (Ehlting et al., 1999; Lauvergeat et al., 2001; Sibout et al., 2005; Eudes et al., 2016).

The apricot mei varieties “Fenghou,” “Dan Fenghou,” “Xiangruibai,” and “Yanxing” are commonly used in landscaping in North China because of their outstanding cold resistance. In terms of the aspect of ornamental characters, the flowers of ‘Fenghou’ have no fragrance to the human nose, and those of “Dan Fenghou” and “Yanxing” have a slight fragrance, whereas “Xiangruibai” is the only apricot mei variety exhibiting the characteristic floral scent of *P. mume*. To clarify the molecular mechanism underlying the lack of a characteristic floral scent in apricot mei varieties, bioinformatics, plant physiology, biochemistry, and molecular biology techniques were comprehensively utilized to comparatively analyze the volatile metabolites in headspace, intracellular pools of fragrance components, enzyme activity and flower transcriptomes of the different varieties of *P. mume*. Our study indicated that the different apricot mei varieties had different mechanisms leading to the lack of the characteristic floral scent. The reduction of benzaldehyde to benzyl alcohol is the key step for limiting the production of the floral scent in the “Fenghou” variety. Low PmBAR1 activity and the low expression level of *PmBAR3* were the main reasons for lack of floral scent in the “Fenghou” variety. In addition, a series of genes that might cause the loss of the characteristic floral scent in apricot mei were identified by integrative analysis of the metabolites and transcriptomes.

## Materials and Methods

### Plant Materials

The blooming flowers of five *P. mume* varieties, “Beijing Yudie,” “Xiangruibai,” “Dan Fenghou,” “Fenghou,” and “Yanxing,” were collected from plants grown in an open field at Beijing Forestry University in China. All five varieties are diploid. They are over twenty years old and bloom during February to March every year. The full blooming flowers with flat petals and fresh pollen were collected. Approximately five flowers of each variety were collected as one sample and immediately frozen in liquid nitrogen. The samples were then stored at −80°C prior to endogenous fragrance component detection, RNA-seq and enzyme activity analysis. Fresh flowers collected on the same day and time were used for headspace analysis of volatiles. Samples from three different plants of each variety were analyzed as three biological replicates.

### Floral Scent Emission and Intracellular Pool Analysis

The floral scent components were determined by GC-MS using the internal standard method, and 5 ng of benzyl propionate was added in each sample and used as an internal standard. The emitted volatiles of fresh flowers were collected in a 100-mL sealed gas bottle in a 30°C water bath for 30 min, and approximately 0.2–0.3 g whole flowers were used and 2.5 ng of benzyl propionate was added as an internal standard. The volatile floral scent compounds were detected using GC-MS, carried out by Shimadzu QP2010 (Shimadzu, Kyoto, Japan) equipped with a DB-5MS capillary column (30 × 0.25 mm, 0.25 μm thickness, Shimadzu, Kyoto, Japan), and a SPME fiber coated with divinylbenzene/carboxen/polydimenthylsiloxane (50/30 μm DVB/CAR/PDMS) was selected. The injection temperature was held at 250°C. The GC oven temperature was started at 40°C for 2 min and then was increased to 250°C at a rate of 5°C/min with a hold for 6 min. More details are provided in a previous study (Zhang et al., 2019a). The intracellular pools of the floral scent components in different varieties were detected as described in a previous study (Bao et al., 2019). The volatile compounds from three different plants of each variety were measured and analyzed.

The peak area of every scent compound was integrated to obtain the total ion current, removing the peaks presented in an empty sample. Individual compounds were tentatively identified by comparing the mass spectra with the NIST11 library (the National Institute of Standards and Technology 2011, Shimadzu, Japan). Main compounds were confirmed by comparison with authentic standard samples.

### Metabolite Relative Emission Efficiency Calculation

The emission efficiencies of different components in the different varieties were calculated by dividing the emission level by the intracellular content. The relative emission efficiency of benzyl acetate in the “Beijing Yudie” variety was 100% and was used as a reference to normalize the relative emission efficiencies of the components in the other varieties.

### Enzyme Activity Analysis in Different Varieties

To compare the enzyme activity levels in the flowers of the five *P. mume* varieties, crude protein samples were extracted using a protein extraction buffer containing 50 mM Tris–HCl (pH 7.5), 150 mM NaCl, 10 mM MgCl_2_, 0.1% NP-40 and a protease inhibitor cocktail (4693159001, Roche). To detect the acyltransferase activity (BEAT and BPBT), 100 μg of crude protein was added to 2 mL of enzyme reaction buffer containing 50 mM Tris–HCl (pH 7.2), 0.5 mM acyl donor (acetyl-CoA for BEAT or benzoyl-CoA for BPBT) and 1 mM benzyl alcohol. The assays were carried out in a 30°C water bath for 1 h. The components were extracted with 0.5 mL of ethyl acetate using a vortex for 15 min, and 5 ng of benzyl propionate was added as an internal standard. The supernatant was collected by centrifugation, and the reaction products (benzyl acetate for BEAT; benzyl benzoate for BPBT) were quantitatively analyzed using GC-MS. The enzyme activity was calculated as the product yield, and a sample containing boiled protein was used as a negative control. Samples from three different plants of each variety were analyzed as three biological replicates.

Since the product coniferyl acetate was unstable and could not be detected by GC-MS, a spectrophotometry method was used to detect CFAT enzyme activity. The reaction buffer and principle have been described previously (Bao et al., 2019; Zhang et al., 2019b). Samples with added boiled protein were used as blank controls. Three biological replicates were analyzed.

To detect BAR enzyme activity, 100 μg of crude protein was added to 2 mL of enzyme reaction buffer containing 20 mM MES (pH 5.7), 200 μM NADH and 1 mM benzaldehyde. The reaction buffer for ADH contained 50 mM Tris-HCl (pH 7.5), 200 μM NAD^+^ and 1 mM benzyl alcohol. The assays were carried out in a 30°C water bath for 1 h, and the products were extracted and quantitatively analyzed by GC-MS as described above. Three biological replicates were analyzed.

### RNA-Seq and Data Analysis

Flower RNA was extracted using an RNAprep Pure Plant Kit (DP432, Tiangen, China). After total RNA was isolated, the RNA integrity, purity and concentration were detected. Three biological replicates for each variety were designed. Sequencing libraries were constructed using an NEBNext Ultra RNA Library Prep Kit for Illumina (NEB, United States), and index codes were added to attribute the sequences to the corresponding samples. mRNA was purified from total RNA using poly-T oligo-attached magnetic beads. Fragmentation was carried out using divalent cations under an elevated temperature in NEBNext First Strand Synthesis Reaction Buffer (5X). First-strand cDNA was synthesized using random hexamer primers and M-MuLV Reverse Transcriptase (RNase H^–^). Second-strand cDNA synthesis was subsequently performed using DNA Polymerase I and RNase H. The remaining overhangs were converted into blunt ends via exonuclease/polymerase activity. After adenylation of the 3′ ends of the DNA fragments, NEBNext Adaptors with hairpin loop structures were ligated to the fragments to prepare them for hybridization. The library fragments were purified to preferentially select cDNA fragments with a length of 150∼200 bp. Then, the size-selected, adaptor-ligated cDNA was incubated with 3 μL of USER Enzyme (NEB) at 37°C for 15 min followed by 5 min at 95°C before PCR. Then, PCR was performed with Phusion High-Fidelity DNA polymerase, universal PCR primers and Index (X) Primer. Finally, the PCR products were purified (AMPure XP system), and the library quality was assessed on an Agilent Bioanalyzer 2100 system. Clustering of the index-coded samples was performed on a cBot Cluster Generation System using a TruSeq PE Cluster Kit v3-cBot-HS (Illumina). After cluster generation, the library preparations were sequenced on an Illumina HiSeq platform, and 125-bp/150-bp paired-end reads were generated. All raw-sequence read data were submitted as a BioProject (PRJNA610678) to the NCBI Sequence Read Archive under accession number SRP251790.

The raw data in fastq format were processed with in-house Perl scripts. In this step, clean data were obtained by removing the reads containing adapter, poly-N or low quality sequences, the Q20 and Q30 values and GC content of the clean data were calculated, and the analyses were performed on clean reads with high-quality sequences. An index of the reference genome was built using Bowtie v2.2.3, and paired-end clean reads were aligned to the reference genome using TopHat v2.0.12. HTSeq v0.6.1 was used to count the reads mapped to each gene. Then, the FPKM of each gene was calculated based on the length of the gene and the number of reads mapped to the gene (Trapnell et al., 2012). Principal component analysis (PCA) was performed using the ggfortify package in R. Differential expression analysis between two varieties was performed using the DESeq R package (1.18.0). DESeq provides statistical routines for determining differential expression in digital gene expression data using a model based on the negative binomial distribution. The resulting *P*-values were adjusted using the Benjamini and Hochberg’s approach for controlling the false discovery rate. Genes identified by DESeq that had adjusted *P*-values < 0.05 were considered differentially expressed. Gene Ontology (GO) enrichment analysis of the differentially expressed genes was performed with the Goseq R package with correction for gene length bias. GO terms with corrected *P*-values < 0.05 were considered significantly enriched for the differentially expressed genes. KEGG Orthology-Based Annotation System (KOBAS) software was used to test the statistical enrichment of the differentially expressed genes in Kyoto Encyclopedia of Genes and Genomes (KEGG) pathways.

Candidate enzyme-coding genes in a genomic database of *P. mume* were identified based on a blastP search in which some reported functional genes were used as query sequences. The extracted protein sequences were confirmed to have conserved domains using CD-Search^1^.

### Integrative Metabolite, Enzyme Activity, and Transcriptome Analysis

Pearson correlation coefficients were calculated for six metabolites and differentially expressed genes (genes with log_2_ fold changes ≥2 or ≤–2). Correlations corresponding to coefficients with *R*^2^ values ≥ 0.8 and *P*-values ≤ 0.01 were selected. The highly associated genes were classified into MapMan bins using the MapCave tool^2^ (Lohse et al., 2014). The relationships between metabolites and transcripts were visualized using Cytoscape (version 3.3.0). Heat maps were drawn using HemI 1.0 software. The correlation coefficients between enzyme activity levels and gene expression levels were calculated with the CORREL function in an Excel spreadsheet.

### qPCR Validation

The total RNA of flowers from five varieties was extracted using previously described methods. First-strand cDNA was synthesized from total RNA using PrimeScriptTM RT Reagent Kit with gDNA Eraser (Takara, Dalian, China) following the manufacturer’s instructions. qPCR was performed on the PikoReal Real–Time PCR System (Thermo Fisher Scientific, China) using the following parameters: 95°C for 30 s, 40 cycles of 95°C for 5 s and 60°C for 30 s, and 60°C for 30 s, concluding with a melting-curve stage for 15 s at 95°C, 1 min at 60°C, and 15 s at 95°C. Each reaction consisted of 1 μL first-strand cDNA, 5 μL SYBR Premix Ex Taq (Takara), 0.1 μL each of 10 μM primer pairs, and 3.9 μL H_2_O. Each sample was assessed in three technical replicates for each of three biological repeats and normalized using *PmPP2A* as an internal control. The transcription levels were determined using the 2^–Δ^ ^Δ^ ^*Ct*^ method. The correlation coefficients between RNA-seq and qPCR were calculated with the CORREL function in an Excel spreadsheet.

### Data Statistical Analysis

Multiple comparisons were used to analyze the significance of the data for floral scent emission efficiency, enzymatic activity and qPCR. The statistical analysis was performed using Origin8 software (OriginLab, Northampton, MA, United States), with one-way ANOVA and Fisher LSD. Different letters indicate a significant difference at the 0.05 level.

### Plasmid Construction

To verify the functions of PmBAR proteins in the different varieties, the coding sequences of *PmBAR1* (Pm012335), *PmBAR2* (Pm013777), and *PmBAR3* (Pm013782) were cloned into the vector pSuper1300 with a Super promoter using the In-Fusion method (In-Fusion HD Cloning Kit, PT5162-1). The coding sequence of *PmBAR1* was amplified using the PmBAR1-F (ACATTTAAATACTAGTatgagcagcggggctggtaa) and PmBAR1-R (TACCGGATCCACTAGTtcagaaattgacaaaattct) primers. *PmBAR2* cDNA was amplified using the PmBAR2-F (ACATTTAAATACTAGTatgcagaactggtactgcta) and PmBAR2-R (TACCGGATCCACTAGTttatttccgcactaaagata) primers. *PmBAR3* cDNA was amplified using the PmBAR3-F (ACATT TAAATACTAGTatgatggagcctgcggtgaa) and PmBAR3-R (TAC CGGATCCACTAGTttaagattgaattttaacgg) primers. Total RNA was isolated from blooming flowers of the “Beijing Yudie” and “Fenghou” varieties and reverse transcribed into cDNA (KR107, Tiangen, Beijing, China), which was then used as a template for gene amplification.

### Enzyme Activity Analysis for PmBARs

To detect PmBAR enzyme activity levels, *PmBAR1*, *PmBAR2*, and *PmBAR3* from the “Xiangruibai” and “Fenghou” varieties were transiently expressed in tobacco leaves by *Agrobacterium tumefaciens*-mediated transformation. Crude protein was extracted from the samples using a protein extraction buffer as described above. Then, 100 μg of crude protein was added to 2 mL of enzyme reaction buffer containing 20 mM MES (pH 5.7), 200 μM NADH and 1 mM benzaldehyde. The assays were carried out in a 30°C water bath, and the increase in absorbance at 340 nm was recorded using a spectrophotometer to determine the consumption of NADH (the enzyme activity). Samples with added boiled protein and without NADH were used as blank controls. Three biological replicates were designed.

### Protein Structure Prediction

The protein tertiary structures of PmBAR2 and PmBAR3 were predicted using SWISS-MODEL^3^. The amino acid sequences of PmBAR2-Xia and PmBAR3-Xia2 were submitted for template identification. PhCCR1 was identified as the best-matched protein (4r1t.1.A) and used as a template for modeling. Structural alignments were performed with 3D Molecule Viewer (Vector NTI Advance 11.0). A model of PhCCR1 NADP^+^ binding was obtained from the Research Collaboratory for Structural Bioinformatics Protein Data Bank^4^ with the accession code 4R1S.

## Results

### Lack of the Characteristic *P. mume* Floral Scent in Varieties of Apricot Mei

**FIGURE 1:**
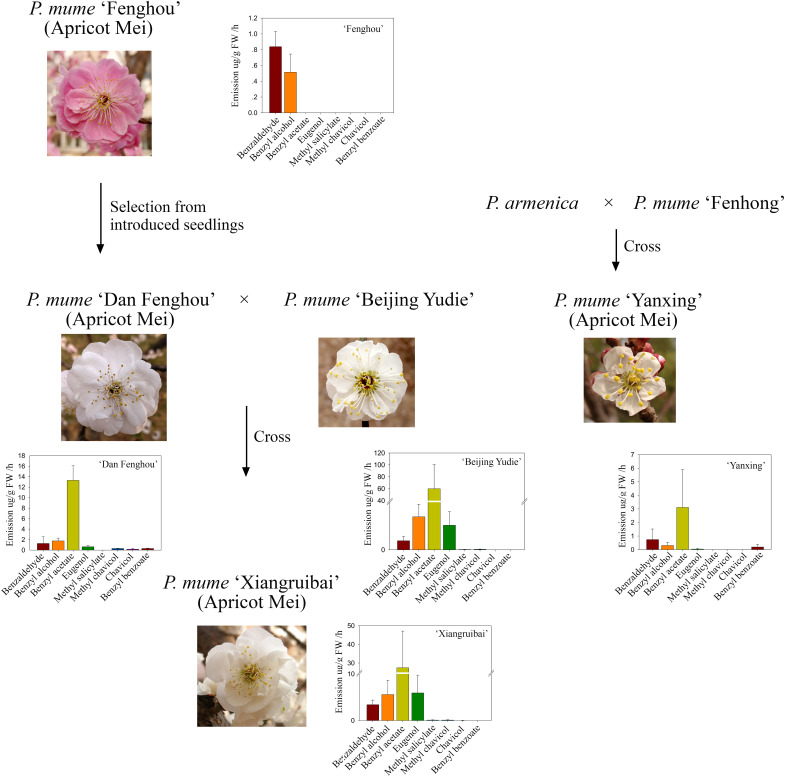
Genetic relationships and floral scent metabolites among the five varieties. The family trees indicate the origins and genetic relationships of five *P. mume* varieties “Fenghou”, “Dan Fenghou”, “Beijing Yudie”, “Xiangruibai” and “Yanxing” The bar graphs indicate the floral scent components detected in the headspace of fresh flowers by HS-SPME-GC-MS. The values on the graphs are presented as the mean values of three biological replicates ± SDs.

The apricot mei variety “Fenghou,” which was introduced from Japan, has no floral fragrance for the human nose. “Dan Fenghou,” which has a slight floral scent and is different from other varieties of *P. mume*, was selected from the natural pollination offspring of “Fenghou” variety. The apricot mei variety “Xiangruibai,” which is the only variety with the characteristic floral scent of *P. mume*, is the inbred product of “Dan Fenghou” and “Beijing Yudie” varieties. Another apricot mei variety “Yanxing,” which has a slight floral scent, is the inbred variety of *Prunus armeniaca* and *P. mume* “Fenhong.” The genetic relationships were shown in Figure 1.

The headspace volatiles of the blooming flowers of these varieties were analyzed. As shown in Figure 1 and Supplementary Table 1, benzyl acetate, benzyl alcohol, benzaldehyde and eugenol were the main volatile components in the fragrant varieties “Beijing Yudie” and “Xiangruibai.” Benzyl acetate was the most abundant component, and its content was highest in the “Beijing Yudie” variety. This level was approximately one-half the level in the “Xiangruibai” variety and one-quarter that in the “Dan Fenghou” variety. Little or no benzyl acetate was detected in the “Yanxing” and “Fenghou” varieties. The differences in eugenol contents among varieties were similar to those of benzyl acetate; its content was higher in the “Beijing Yudie” and “Xiangruibai” varieties and lower in the “Dan Fenghou” and “Yanxing” varieties, and no eugenol was detected in “Fenghou” variety. The benzyl alcohol content was highest in the fragrant varieties “Beijing Yudie” and “Xiangruibai.” The differences in benzaldehyde content among varieties were the same as those of benzyl alcohol. Methyl salicylate was specific to the “Beijing Yudie” and “Xiangruibai” varieties when compared to the “Dan Fenghou,” “Fenghou,” and “Yanxing” varieties. Chavicol was the specific component in the “Xiangruibai” and “Dan Fenghou” varieties. Benzyl benzoate was specific to the apricot mei varieties “Dan Fenghou” and “Yanxing.” However, methyl chavicol was detected mainly in the fragrant varieties “Beijing Yudie,” “Xiangruibai,” and “Dan Fenghou” and none was detected in the low or non-fragrant varieties “Yanxing” and “Fenghou.” Combined with the abundance and odor perception thresholds of the components (Sánchez-Palomo et al., 2010), benzyl acetate and eugenol were considered to be the main components of the characteristic floral scent of *P. mume*. Fewer or no benzyl acetate and eugenol were detected in apricot mei varieties “Dan Fenghou,” “Yanxing,” and “Fenghou.”

### The Emission Efficiencies of the Floral Scent Components Were Different Among Varieties

**FIGURE 2:**
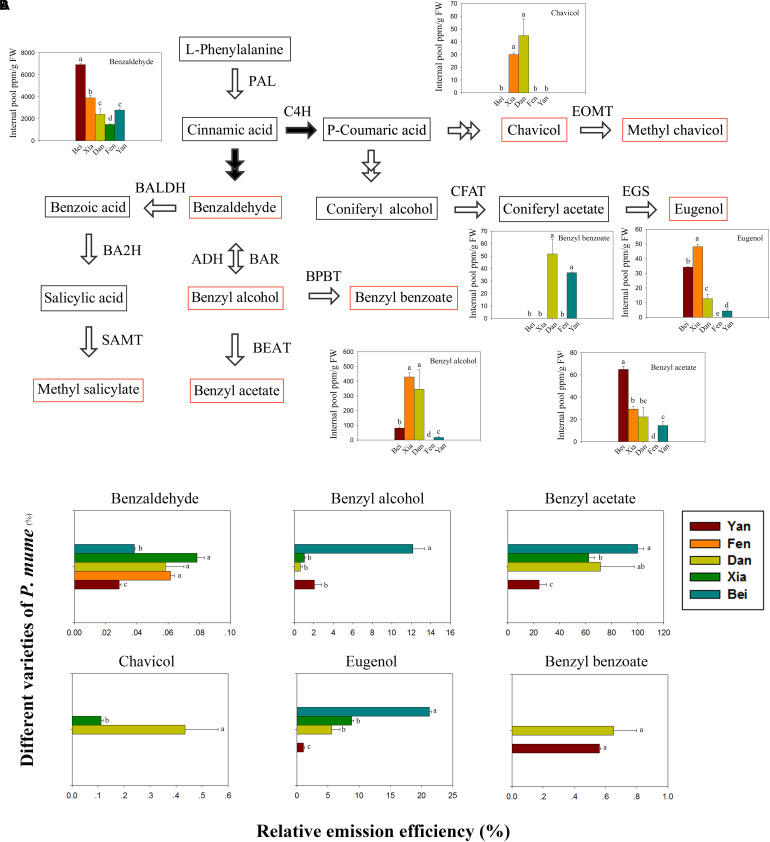
Floral scent biosynthetic pathway and intracellular pools and emission efficiencies of fragrance components. **(A)** The floral scent biosynthetic pathway in *P. mume* is shown. The black arrows indicate the starting steps of the *trans*-cinnamic acid and *para*-coumaric acid pathways. The red rectangles indicate the metabolites that were detected in the headspace. The colored graphs show the intracellular pools of floral scent metabolites. The data are presented as the mean values of three biological replicates ± SDs. Different letters indicate significant differences at the *P* = 0.05 level. **(B)** The relative emission efficiencies of different components in the different varieties. The data are presented as the mean values of three biological replicates ± SDs.

The floral scent components in the intracellular pools were detected in five varieties. The phenylpropanoid/benzenoid biosynthetic pathway is divided into the *trans*-cinnamic acid pathway and *para*-coumaric acid pathway (Colquhoun et al., 2010). As shown in Figure 2A, benzaldehyde, benzyl alcohol, benzyl acetate, methyl salicylate, and benzyl benzoate are derived from *trans*-cinnamic acid, whereas eugenol, chavicol and methyl chavicol are derived from *para*-coumaric acid. Six types of components were detected (e.g., benzaldehyde, benzyl alcohol, benzyl acetate, benzyl benzoate, eugenol, and chavicol) in the intracellular pools of five varieties. However, methyl salicylate and methyl chavicol, which were identified in the headspace, were not detected. The benzaldehyde content was highest in the intracellular pools and was followed by benzyl alcohol. When comparing the varieties, the benzaldehyde and benzyl acetate contents were highest in “Beijing Yudie,” followed by “Xiangruibai,” while the content of benzyl alcohol and eugenol was highest in “Xiangruibai.” Consistent with the results of the headspace volatiles detection, benzyl benzoate was detected only in “Dan Fenghou” and “Yanxing,” while chavicol was detected only in “Xiangruibai” and “Dan Fenghou.” Compared with those in the fragrant varieties, the benzaldehyde and eugenol contents were lower in “Dan Fenghou;” the benzyl acetate content was slightly lower than that in “Xiangruibai;” however, the benzyl alcohol content was even higher than that in “Beijing Yudie.” Compared with those in the fragrant varieties, the contents of benzaldehyde, benzyl alcohol, benzyl acetate and eugenol were lower in “Yanxing.” Only benzaldehyde was detected in the fragrance-free variety “Fenghou,” in which the benzaldehyde content was lowest compared with the other varieties.

The relative emission efficiencies of each metabolite in different varieties were then analyzed (Figure 2B). We know that the emission efficiencies differ for different types of metabolites, which may partly depend on their physical properties. The emission efficiency of benzyl acetate was highest, while that of benzaldehyde was lowest. The efficiency of benzyl acetate was 25 times higher than that of benzaldehyde in “Beijing Yudie.” We also found that, except for benzyl benzoate, the emission efficiencies of the metabolite types were different among different varieties. As the main floral scent components of *P. mume*, the emission efficiencies of benzyl acetate and eugenol were highest in “Beijing Yudie,” while they were lowest in “Yanxing.” The results indicated that emission efficiency is also an important factor affecting the flower fragrance intensity of *P. mume* varieties.

### Different Steps in the Pathway Limit Floral Scent Synthesis in Different Varieties of Apricot Mei

**FIGURE 3:**
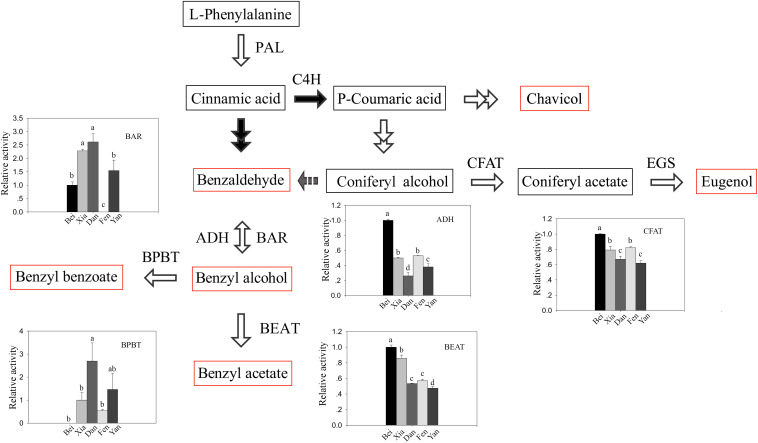
The activity of key enzymes in the floral scent biosynthetic pathway. In the floral scent biosynthetic pathway, the red rectangles indicate the metabolites that were detected in intracellular pools, and the gray arrow indicates a proposed step that has not previously been described. The black-and-white graphs show the key enzyme activity levels in the different varieties. The data are presented as the mean values of three biological replicates ± SDs. Different letters indicate significant differences at the *P* = 0.05 level.

Since the contents of benzaldehyde in the intracellular pools were abundant even in the “Fenghou” variety (Supplementary Table 2), the activity levels of key enzymes downstream of the floral scent synthesis pathway were compared among five varieties. As shown in Figure 3, the activity levels of BAR, ADH, BEAT, and BPBT among the different varieties were all positively correlated to the intracellular metabolite levels. The BAR activity was higher in the “Xiangruibai” and “Dan Fenghou” varieties followed by the “Beijing Yudie” and “Yanxing” varieties; none was detected in the fragrance-free variety “Fenghou.” As a reverse reaction to BAR, ADH activity could be detected in all of five varieties. The results indicated that lack of BAR activity was the key reason limiting the benzyl acetate synthesis in the “Fenghou” variety, and ADH activity was also an important factor limiting the accumulation of benzyl alcohol. BEAT activity was higher in varieties displaying the characteristic fragrance, namely, “Beijing Yudie” and “Xiangruibai,” followed by the low and no fragrance varieties “Dan Fenghou,” “Yanxing,” and “Fenghou.” It is suggested that the large amount of benzyl acetate synthesis in the “Beijing Yudie” variety is dependent on high BEAT activity, although benzyl alcohol in the intracellular pools was not enriched in this variety. BPBT activity was only significantly detected in the “Dan Fenghou” and “Yanxing” varieties. Moreover, relatively high CFAT activity was detected in the “Dan Fenghou” and “Yanxing,” varieties, especially the “Fenghou” variety, although fewer or no eugenol could be detected either in the intracellular pools or in the emissions. The results indicated that CFAT was not the key step affecting the synthesis of eugenol in apricot mei. Additionally, it was interesting to find a new phenylpropanoid metabolism pathway in *P. mume* from coniferyl alcohol to benzaldehyde; however, the enzyme catalyzing this reaction remains unknown.

### Analysis of Differentially Expressed Genes in the Flowers of Five Different Varieties by RNA-Seq

**FIGURE 4:**
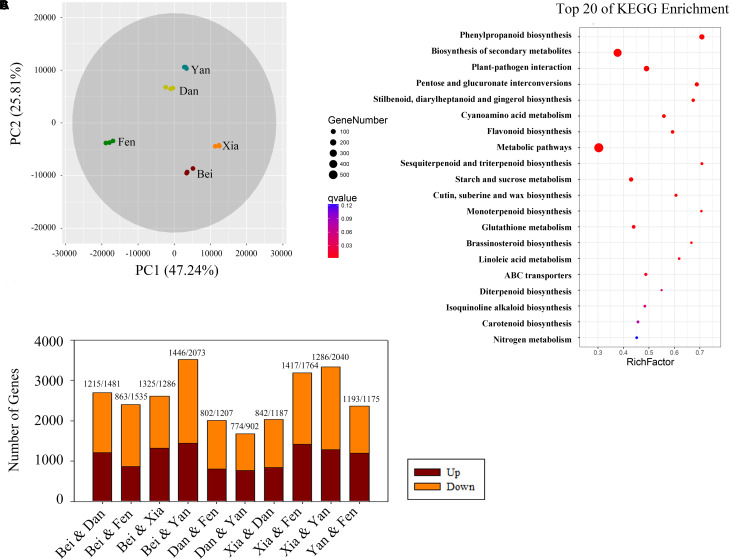
Bioinformatics analysis of differentially expressed genes in the transcriptomes of the five varieties. **(A)** PCA score plot for the five varieties. **(B)** Numbers of differentially expressed genes (genes with a fold change > 4) between each pair of varieties. The numbers of upregulated and downregulated genes are shown above the columns. **(C)** Top 20 pathways in the KEGG enrichment analysis. The number of genes in each category is equal to the point size. The point color represents the *q*-Value.

To identify the different expression levels of genes in the floral metabolism of different *P. mume* varieties, RNA isolated from the blooming flowers of the five varieties was sequenced and generated 739 million clean reads representing 110.87 Gbp; the average error rate of the sequenced bases was less than 0.02% (Supplementary Table 3). The genome mapping information for each sample is displayed in Supplementary Table 4. The percentage of total mapping was between 82.93 and 90.59%. The Pearson correlation coefficients (*R*^2^) among biological replicates were greater than 0.993 for each sample, indicating good repeatability and a reliable dataset (Supplementary Figure 1). Eight hundred thirty-four novel genes and 12,807 novel isoforms were obtained (Supplementary Tables 5, 6). PCA of the gene expression showed that the five cultivars were clearly separated in the PC1 × PC2 score plots (Figure 4A). The first principal component (PC1, 47.24% of the total variables) was clearly separated between the “Fenghou” and “Xiangruibai” varieties, while the differences among the “Beijingyudie,” “Dan Fenghou,” and “Yanxing” varieties resulted from PC2 (25.81% variables).

Analysis of the flower transcriptomes showed that a total of 28,702 transcripts were identified from the five varieties. Figure 4B shows the numbers of the differentially expressed genes between the two varieties (fold change > 4). The number of differentially expressed genes was highest between the “Beijing Yudie” and “Yanxing” varieties, with 1,446 upregulated and 2,073 downregulated genes, while it was lowest between the “Dan Fenghou” and “Yanxing” varieties, with 774 upregulated and 902 downregulated genes, respectively. A total of 7,508 differentially expressed genes were obtained from the five varieties of *P. mume*. GO significant enrichment analysis showed that these differentially expressed genes were mainly involved in “metabolic process,” “binding,” “catalytic activity,” and “membrane” among the biological process, molecular function and cellular component categories (Supplementary Figure 2). Then, KEGG pathway enrichment analysis divided the differentially expressed genes into five groups: “metabolism,” “genetic information processing,” “environmental information processing,” “cellular processing,” and “organismal systems” (Supplementary Figure 3). The largest group was “metabolism,” which included 621 genes, followed by “genetic information processing.” The top 20 pathways in the KEGG enrichment pathway analysis are shown in Figure 4C, in which the phenylpropanoid biosynthesis pathway was the most significant. These results indicated that the differentially expressed genes in five varieties of *P. mume* were mainly enriched in metabolism and enzyme catalysis, especially in the phenylpropanoid biosynthesis pathway.

### Analysis of Differentially Expressed Genes in the Floral Scent Metabolic Pathway Among the Five Different Varieties

**FIGURE 5:**
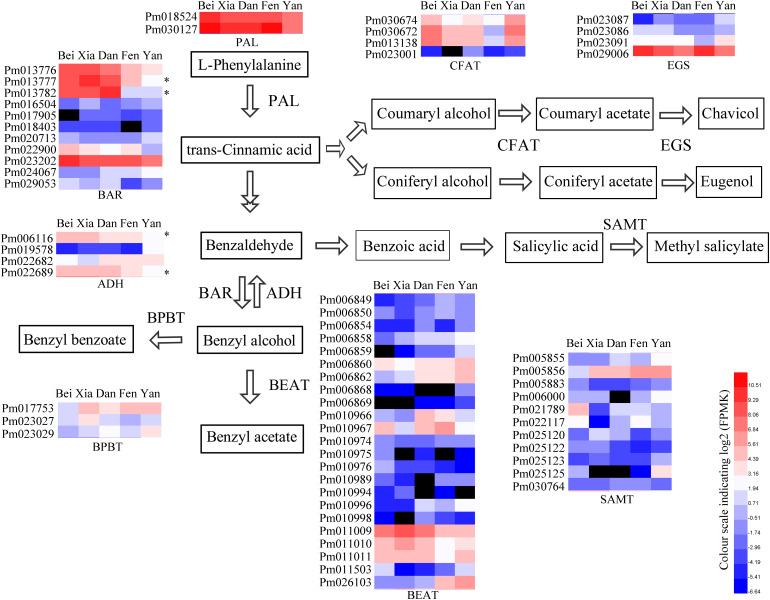
Heat maps of the differential expressed genes encoding key enzymes in the floral scent biosynthetic pathway in the five varieties. The key floral scent biosynthetic pathway in *P. mume* is shown. In the heat maps, red and blue represent high and low transcript expression, respectively, and black represents undetectable expression. The asterisks indicate the genes with strong positive correlations between gene expression and enzyme activity.

To identify the key genes that affect floral scent synthesis in different varieties, the differential expression profiles of key enzyme genes in floral scent metabolism were analyzed among five varieties (Figure 5). The genes were identified through blastP and conserved domain analyses based on some reported functional genes (Supplementary Tables 7, 8). The expression level of *PAL*, which is responsible for the first step of phenylpropanoid metabolism, was higher in the “Beijing Yudie” variety than the apricot mei varieties. Conversion of benzaldehyde into benzyl alcohol is the key limiting step in benzyl acetate synthesis in the “Fenghou” variety, and *PAR* was selected as the query gene to search for candidate *BAR* genes in the *P. mume* genome. Eleven *BAR* genes showed differential expression among the five varieties, including two genes that had relatively strong positive correlations with BAR enzyme activity in the five varieties. xylB was used as the query to search for candidate *ADH* genes in the *P. mume* genome. Four *ADH* genes showed differential expression among the five varieties, including two genes, Pm0061116 and Pm022689 which were strongly positively correlated with ADH activity. Twenty-three *PmBEAT* genes were identified to be differentially expressed among the five varieties, including *PmBEAT36* (Pm011009) and *PmBEAT37* (Pm011010), the enzyme activity of which has been verified *in vitro* and *in vivo* (Bao et al., 2019). The expression levels of *PmBEAT36* and *PmBEAT37* were relatively higher in the “Beijing Yudie,” “Xiangruibai,” and “Dan Fenghou” varieties than in the other varieties. Three *BPBT* genes were identified, one of which had higher expression levels in apricot mei varieties than in the “Beijing Yudie” variety. In addition, four *CFAT* and *EGS* genes that were differentially expressed among the five varieties were also identified.

### Enzyme Activity of PmBAR Proteins for Different Varieties of *P. mume*

**FIGURE 6:**
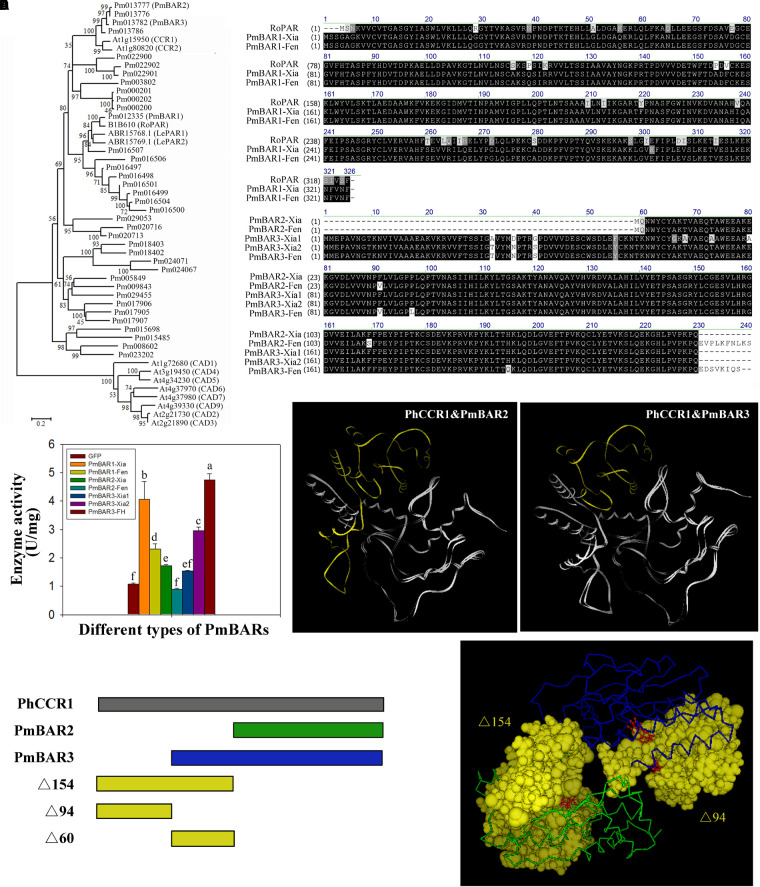
Comparison of the sequences and enzyme activity of PmBARs from the different varieties. **(A)** Comparison of the PmBAR amino acid sequence in the “Xiangruibai” and “Fenghou” varieties. RoPAR, PAR in rose. RoPAR was used as a reference sequence to identify PmBARs in *P. mume*. **(B)** Phylogenetic tree of PmBARs, PARs, CCRs and CADs. The tree was generated using MEGA 6 software with the neighbor-joining method. The bootstrap values (%) are shown next to the branches. The branch lengths were determined by the evolutionary distance. **(C)** Enzyme activity of PmBARs from the “Xiangruibai” and “Fenghou” varieties. The data are presented as the mean values of three biological replicates ± SDs. The data are presented as the mean values of three replicates ± SDs. A sample expressing GFP was used as a negative control. **(D)** Comparison of protein tertiary structures between PhCCR1 and PmBARs. The underlying N-terminal deletion sequences PmBARs compared with PhCCR1 are marked in yellow. PhCCR1, CCR1 in petunia. **(E)** Model of NADP^+^ binding to the N-terminus of PhCCR1 (PDB ID: 4R1S). Schematic diagram of the protein structures of PmBARs and PhCCR1 (left), in which different colors reflect the different segments marked in the tertiary structure (right). NADP^+^ is shown in red and in a ball-and-stick representation.

The reduction of benzaldehyde to benzyl alcohol is the limiting step for the synthesis of benzyl acetate in the “Fenghou” variety. Both PAR and CCR belong to the SDR family, and CAD is also an enzyme that catalyzes the conversion between aldehydes and alcohols. Therefore, some CCRs, CADs identified in *Arabidopsis* and PARs in rose and tomato were selected to construct the phylogenetic tree with BAR in *P. mume*. As shown in Figure 6A, Pm012335 clustered with RoPAR, LePAR1 and LePAR2; however, there was no significant difference in *PmBAR1* gene expression among the five varieties. In contrast Pm013777 and Pm013782 were clustered with CCR in the phylogenetic tree, but the expression levels of these genes had relatively strong positive correlations with BAR activity in the five varieties. Based on the phylogenetic tree and gene expression patterns, these three candidate genes were selected and named *PmBAR1*, *PmBAR2*, and *PmBAR3*, respectively.

The 3 *PmBAR* genes in the fragrant variety “Xiangruibai” and non-fragrant variety “Fenghou” were cloned (Supplementary Figures 4–6). As shown in Figure 6B, the correspondence of the amino acid sequences between PmBAR1 and RoPAR was greater than 88%. A difference was detected only in the 302nd amino acid between the sequences of *PmBAR1-Xia* and *PmBAR1-Fen*, both of which were found in the “Xiangruibai” variety (allelic heterozygosity). Enzyme activity analysis showed that significantly higher activity of PmBAR1-Xia than PmBAR1-Fen (Figure 6C), which indicated that the 302K site played an important role in the activity of PmBAR1.

Comparing the amino acid sequence of PmBAR2 with that of PmBAR3, a 60-amino-acid sequence was missing from the N-terminus of PmBAR2, except for the initial two amino acids; the PmBAR2-Xia sequence was the same as that of PmBAR3-Xia2 except for the missing N-terminus. The PmBAR2-Xia activity was weaker than that of PmBAR3-Xia2 (the activity of PmBAR2-Fen was not even detected), which suggested that the missing Δ60 in the N-terminus of PmBAR2 played an important role in enzyme activity.

Eight amino acid differences were identified between the sequences of PmBAR3-Xia1 and PmBAR3-Xia2, while comparing PmBAR3-Xia2 with PmBAR3-Fen, three amino acid differences and a C-terminal deletion were found. The activity of PmBAR3-Fen was higher than that of PmBAR3-Xia2, and the activity of PmBAR3-Xia1 was even lower than that of PmBAR3-Xia2. The differences in the sequences of these three alleles are potential sites affecting PmBAR3 activity.

The protein structures of PmBAR2 and PmBAR3 were modeled by bioinformatics according to the tertiary structure of PhCCR1 in petunia. It was found that both PmBAR2 and PmBAR3 had N-terminal deletions (e.g., a 154-aa deletion for PmBAR2 and a 94-aa deletion for PmBAR3) compared with PhCCR1 (Figure 6D and Supplementary Figure 7). It is known that the N-terminal segment of the CCR protein can recognize the substrate NADPH and that Arg-38, Lys-44 are key residues for distinguishing between NADPH-specific SDRs from NADH-specific SDRs (Filling et al., 2002; Pan et al., 2014). It was interesting that the activity of PmBAR3 was not significantly different when using NADPH or NADH as substrates (data not shown). The NADP^+^-binding spatial structures of the Δ154 and Δ94 segments were determined using the PhCCR1 structure as a model. As shown in Figure 6E, the Δ154 segment contained most of the NADP^+^-binding pocket, while the Δ94 segment was only part of the pocket. It is speculated that the Δ94 deletion of PmBAR3 causes it to lose specificity for using NADPH as a substrate, while the Δ154 deletion of PmBAR2 causes it to lose binding ability to NADPH, which leads to a decrease in enzyme activity. The above results indicate that PmBAR1 and PmBAR3 are the main enzymes affecting the reduction of benzaldehyde to benzyl alcohol in *P. mume*. In addition, the low activity of PmBAR1-Fen and the low expression level of *PmBAR3* are the main limits for benzyl alcohol synthesis in “Fenghou.”

### Correlation Analysis Between Transcripts and Floral Scent Metabolites Reveals Differential Regulatory Networks in the Different Varieties

**FIGURE 7:**
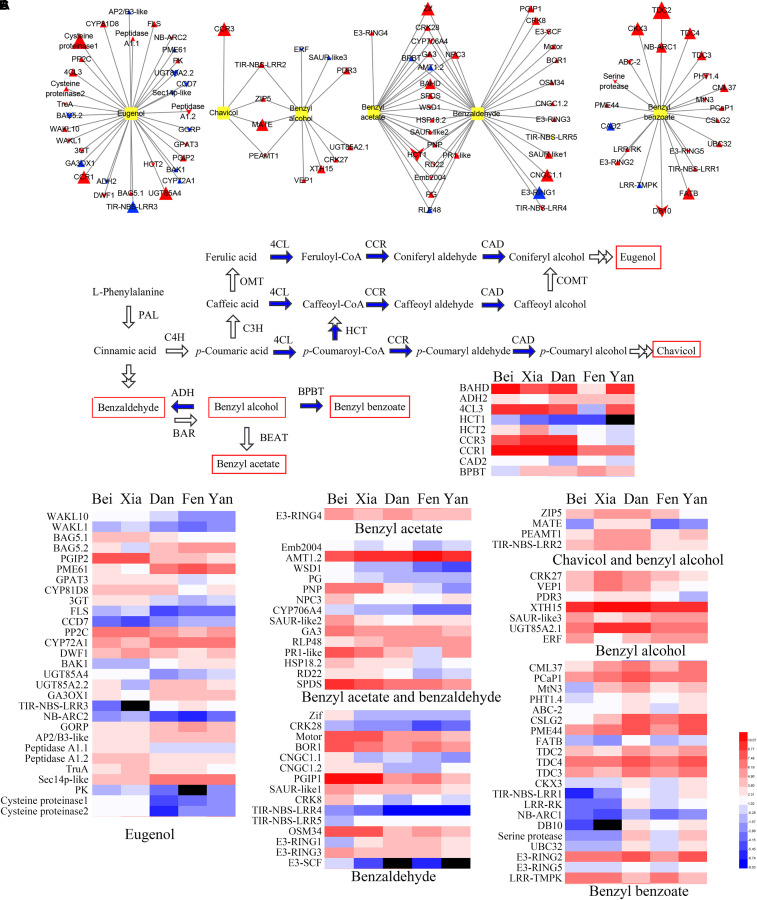
Connections between genes and floral scent metabolites and heat maps of gene expression. **(A)** The connections between highly associated genes and six metabolites were assessed by correlation analysis. Yellow boxes indicate metabolites. Triangles indicate upregulated genes in blooming flowers, while chevrons indicate downregulated genes in blooming flowers. The sizes of the triangles and chevrons represent the log_2_ (fold change) ratios in flowers to buds. Red symbols indicate positive correlations, and blue symbols indicate negative correlations. **(B)** Biosynthesis pathway and heat maps of the expression of the highly correlated enzyme-coding genes. The blue arrows indicate steps for which the enzyme-coding genes were identified in the correlation analysis. Red rectangles indicate the metabolites detected in intracellular pools. **(C)** Heat map of the expression of other genes (non-enzyme-coding genes) that were highly correlated with the six metabolites. In the heat maps, the values were normalized by log2 transformation. Red and blue represent high and low transcript expression, respectively. Black represents undetectable expression.

To further understand the different regulatory networks of volatile phenylpropanoids among the varieties, we analyzed the correlations between metabolites and transcripts in the five varieties. Previous transcriptome data from *P. mume* flower buds and blooming flowers (using the “Sanlun Yudie” variety as material, with a similar floral scent composition and release pattern to the “Beijing Yudie” variety) (Zhao et al., 2017) were also used to screen 1,444 transcripts from among 7,508 transcripts with at least four-fold differential expression between the two stages. The correlation analysis between six components detected in the intracellular pools and the 1,444 differentially expressed transcripts showed that 158 transcripts were highly correlated with metabolites (Supplementary Table 9). These transcripts were classified into 14 groups depending on their annotated functions and included 103 upregulated and 55 downregulated genes in blooming flowers compared with buds (Table 1). Based on these results, a network of the interactions between the six metabolites and 100 transcripts (transcripts in the categories of “misc” and “not assigned” were not included) was created (Supplementary Table 10). The expression profiles of the different varieties and gene annotation are shown in Supplementary Table 11. As shown in Figure 7A, 33, 5, 11, 20, 32, and 22 genes were correlated with the floral scent components eugenol, chavicol, benzyl alcohol, benzyl acetate, benzaldehyde and benzyl benzoate, respectively; among these genes, four were correlated with both benzyl alcohol and chavicol, and 19 genes were correlated with both benzaldehyde and benzyl acetate. In total, 79 genes and 21 genes were positively and negatively associated with metabolites, respectively.

**TABLE 1:** Functional categories of the differentially expressed genes that were highly correlated with metabolites.

			Regulated in bud & flower	
BIN code	Annotation	Gene number	Up	Down
BIN9	Mitochondrial ATP synthesis	1	0	1
BIN10	Cell wall	8	8	0
BIN11	Lipid metabolism	5	3	2
BIN16a*	Floral scent metabolism	9	6	3
BIN16b	Other secondary metabolism	7	5	2
BIN17	Hormone metabolism	15	11	4
BIN20/22/24	Stress and defense	16	11	5
BIN26	Misc	24	13	11
BIN27	Regulation of transcription	4	3	1
BIN29	Protein	14	8	6
BIN30	Signaling	7	6	1
BIN31/33	Cell organization and development	6	3	3
BIN34	Transport	9	7	2
BIN35	Not assigned	34	19	15
Total	–	158	103	55

*BIN16a includes some genes in fermentation (ADH), lipid metabolism (4CL), phenylpropanoid metabolism (BAHD, HCT, CCR, and CAD), and stress (BPBT).

Enzymes were positioned mostly downstream of the regulatory network of floral scent synthesis. Nine floral scent-related enzymes were correlated with metabolites (Figure 7B). The homologous genes of *4CL3*, *CCR1*, and *HCT2* were found to be positively correlated with eugenol, whereas the homologous gene of *CCR3* was positively correlated with chavicol. Moreover, the enzyme HCT catalyzes the conversion of coumaroyl-CoA into caffeoyl-CoA, which directs metabolic flux from chavicol synthesis to eugenol synthesis. It was interesting that the expression levels of *HCT2* in the “Beijing Yudie” and “Xiangruibai” varieties were higher than those in the other varieties, which might have led to increases in eugenol production in the “Beijing Yudie” and “Xiangruibai” varieties. Benzyl alcohol is a common substrate for the synthesis of benzyl benzoate and benzyl acetate, and the homologous gene of *BPBT* was negatively correlated with both benzyl acetate and benzaldehyde in this study; furthermore, its expression level was lower in the “Beijing Yudie” variety than the apricot mei varieties. A homologous gene of *BAHD* was also found to be associated with both benzyl acetate and benzaldehyde; however, the step catalyzed by this gene is unknown. The expression levels of *4CL3*, *HCT2*, *CCR1*, *CCR3*, *BPBT*, and *BAHD* were increased in blooming flowers, while *HCT2* was not.

Several hormones may regulate flower fragrance metabolism. For example, ABA and BR may play important roles in the regulation of eugenol synthesis. As shown in Figure 7C, the ABA synthesis-related gene *CCD7*, which was downregulated in blooming flowers, was negatively correlated with eugenol, while *PP2C*, a negative regulator of ABA signaling that was upregulated in blooming flowers, was positively correlated with eugenol. Two of three BR synthesis- and signal transduction-related genes, *CYP72A1* and *BAK1*, which were upregulated in blooming flowers, were negatively associated with eugenol, while *DWF*1, which was downregulated in blooming flowers, was positively associated with eugenol. CK may play an important role in regulating the synthesis of eugenol, chavicol, benzyl alcohol and benzyl benzoate. Two UDP-glucosyl transferases involved in CK degradation, UGT85A4 and UGT85A2.2, were highly correlated with eugenol. UGT85A4, which was upregulated in blooming flowers, was positively correlated with eugenol, while UGT85A2.2, which was downregulated in blooming flowers, was negatively correlated with eugenol. Another UDP-glucosyl transferase, UGT85A2.1, which was upregulated in blooming flowers, was positively correlated with benzyl alcohol. The homologous gene of *CKX3*, which was upregulated in blooming flowers, was positively correlated with benzyl benzoate. Ethylene, GA and auxin may also play important roles in floral scent metabolism. A homologous gene of *ERF* that was downregulated in blooming flowers was negatively associated with benzyl alcohol. A homologous gene of *GA3OX1* that was upregulated in blooming flowers was negatively associated with eugenol, while the homologous gene of *GA3*, which was upregulated in blooming flowers, was positively correlated with both benzaldehyde and benzyl acetate. Three SAUR-like auxin-responsive proteins, which were all upregulated in blooming flowers, were highly associated with benzyl acetate, benzaldehyde and benzyl alcohol.

Two transcription factors were identified to be associated with floral scent in the five varieties. One, an AP2/B3-like transcription factor that was upregulated in blooming flowers, was negatively associated with eugenol. The other, a zinc finger transcription factor Zif that was upregulated in blooming flowers, was positively associated with benzaldehyde.

Posttranslational modification of proteins, especially ubiquitin-mediated degradation, may play an important role in the regulation of floral scent metabolism. Two E3 ubiquitin-protein ligases, E3-RING3 and E3-SCF, were positively correlated with benzaldehyde, while another, E3-RING1, was negatively correlated with benzaldehyde. Three ubiquitylation-related factors, including UBC32, E3-RING2 and E3-RING5, were positively associated with benzyl benzoate. E3-RING4, which was upregulated in blooming flowers, was positively associated with benzyl acetate.

Several stress- and defense-related genes may also play important roles in flower fragrance metabolism, including *HSP18.2*, *RD22*, *SPDS*, and *R* genes. There may be some overlap between the plant stress response and floral scent metabolism networks, since most floral fragrance components can inhibit pathogen growth.

Transporters may also play important roles in floral scent metabolism. It has been reported that some membrane transporters can affect the biosynthesis and emission of phenylalanine-derived floral scent metabolites (Widhalm et al., 2015; Adebesin et al., 2017). Nine transporters for metal ions, phosphates and drugs, namely, AMT1.2, PHT1.4, ZIP5, PDR3, ABC-2, BOR1, CNGC1-1, CNGC1-2, and MATE were identified as being associated with floral scent metabolites.

To validate the expression of the genes, qPCR was performed for 10 randomly selected genes with gene-specific primers (Supplementary Table 12). The correlations in the gene expression levels between the RNA-seq and qPCR results were analyzed for the five varieties (Supplementary Table 13). The results showed a high degree of correlation between the datasets. As shown in Supplementary Figure 8, *PmBEAT3*6, *SERK2*, *E3-RING4* and *E4* were highly expressed in the varieties “Beijing Yudie,” “Xiangruibai,” and “Dan Fenghou,” whereas *BPBT*, *SAMT1*, *BAK1*, and *UGT71B8* were highly expressed in the low-fragrance or odorless varieties “Dan Fenghou,” “Fenghou,” and “Yanxing.” *SUMO2* and *E3-RING5* were highly expressed in the “Yanxing” variety.

## Discussion

In this work, the molecular mechanisms underlying the lack of characteristic floral scent in apricot mei were revealed via comprehensive analysis of the differences in volatile and intracellular pools of floral scent metabolites, the activity of key enzymes in the floral scent synthesis pathway, and transcriptome gene expression data among five varieties of *P. mume*. Upon comparison of the volatile metabolites in the different varieties, benzyl acetate and eugenol were determined to be the main components of the characteristic floral scent of *P. mume*. Comparison of the ratios of volatiles and intracellular pool contents of floral scent metabolites revealed that the emission efficiencies of the metabolites were different among the five varieties. The emission efficiencies of benzyl acetate and eugenol were highest in the ‘Beijing Yudie’ variety. It was recently reported that floral volatiles can be actively emitted by an ABC transporter in petunia (Adebesin et al., 2017). The transporters can emit the volatiles out of cells across the plasma membrane and prevent the volatiles from accumulating to toxic levels in cells, which may affect the characteristic floral scent emission efficiencies in *P. mume*. The results of the correlation analysis between metabolites and transcripts also identified some transporters. It is suggested that the transporters may be an important factor affecting the intensity of floral scent in apricot mei. Through analysis of the correlations between floral scent metabolites and transcriptome data, we also identified some highly correlated genes that may affect the synthesis of floral scent. The different expression levels of these genes may underlie the diversity of floral scent metabolism networks among different varieties of *P. mume*.

**FIGURE 8:**
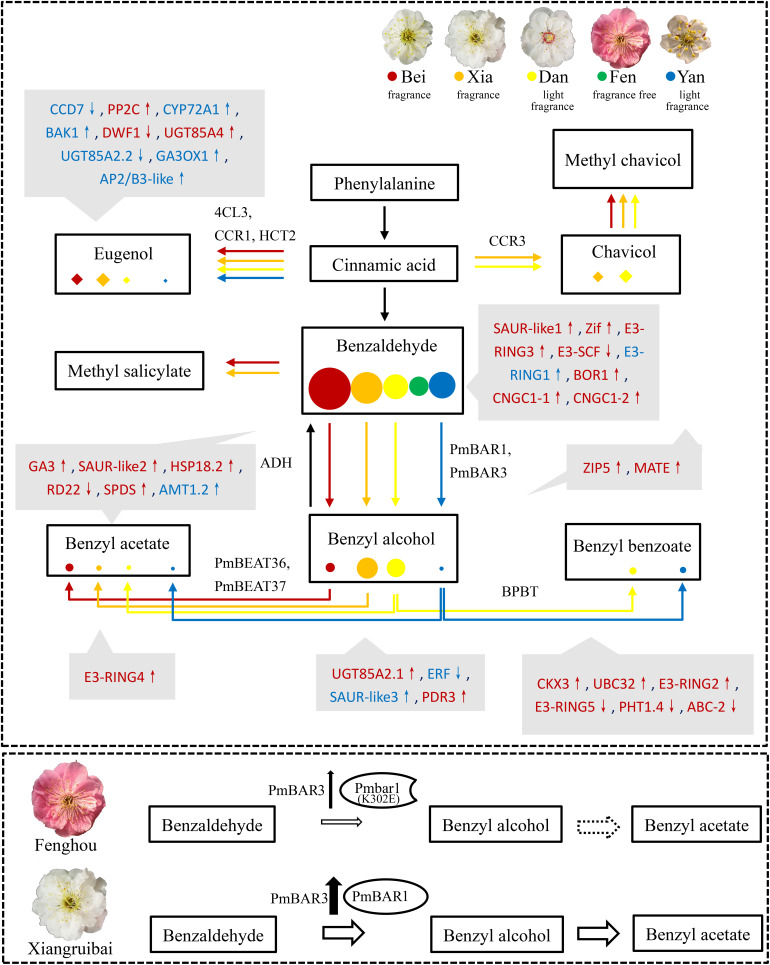
Summary of metabolic flux and differential regulation in the floral scent synthesis pathways of the five varieties. The synthesis pathways of eugenol and benzyl acetate are separated into two metabolic fluxes. The highly correlated genes that may affect the synthesis of floral scent metabolites are shown in gray boxes. Red and blue indicate positively and negatively correlated genes, respectively. Hormones-related genes: CCD7, PP2C, CYP72A1, BAK1, DWF1, UGT85A2.1, UGT85A2.2, UGT85A4, GA3, GA3OX1, SAUR-like1 SAUR-like2, SAUR-like3, ERF, CKX3. Transcript factors: AP3/B3-like, Zif. Ubiquitin modification-related genes: E3-SCF, E3-RING1, E3-RING2, E3-RING3, E3-RING4, E3-RING5, UBC32. Stress-related genes: HSP18.2, RD22, SPDS. Transporters: BOR1, CNGC1-1, CNGC1-2, AMT1.2, ZIP5, MATE, PDR3, PHT1.4, ABC-2. Upward arrows indicate upregulated genes in blooming flowers, while downward arrows indicate downregulated genes in blooming flowers. The enzymes catalyzing the corresponding reaction are beside the arrows. The sizes of the colored circles and diamonds represent the abundances of metabolites in intracellular pools in the five varieties. A working model of the PmBAR-related mechanism underlying the benzyl acetate synthesis dysfunction in the “Fenghou” variety is shown in the image below. The size of the arrow represents the content, and the dotted line indicates non-existence.

Focusing on the limiting step of floral scent synthesis in the “Fenghou” variety, we identified key genes corresponding to BAR activity. RoPAR, which catalyzes the reduction of phenylacetaldehyde to phenylethanol has been identified in rose; this enzyme can also catalyze the reduction of benzaldehyde to benzyl alcohol, albeit with weaker activity (Chen et al., 2011). Rose and *P. mume* both belong to the Rosaceae family. Phenylethanol is the main floral scent component of rose; however, no phenylacetaldehyde or phenylethanol was detected in *P. mume*. We compared the differences in enzyme activity in *P. mume* crude protein extracts using benzaldehyde and phenylacetaldehyde as substrates and found that they were the same as those exhibited by RoPAR. Therefore, it is possible that the orthologous gene of *RoPAR* (*PmBAR1*) is responsible for the reduction of benzaldehyde to benzyl alcohol in *P. mume*. Interestingly, the CCR homologous gene *PmBAR3* also catalyzed the reduction of benzaldehyde to benzyl alcohol. Furthermore, the sequences and activity of PmBARs in different varieties were analyzed, and it was found that a difference in a single residue of PmBAR1-Fen (Pmbar1, K302E) resulted in a decrease in enzyme activity, while the expression level of *PmBAR3-Fen*, which encodes a high-activity enzyme, was quite low in the “Fenghou” variety (FPKM < 3). These factors together caused the low BAR activity and lack of benzyl acetate synthesis in this variety (Figure 8). Furthermore, the additional 60-aa segment at the N-terminus of PmBAR3 compared with PmBAR2 was found to be very important for enzyme activity, and the lack of a 94-aa segment at the N-terminus of PmBAR3 compared with PhCCR1 caused PmBAR3 to lose specificity for the NADPH substrate. Comparison of the variation sites of different PmBAR3 alleles revealed that these sites were different from sites reported to affect substrate recognition and CCR catalytic activity, except for 185V (Supplementary Figure 7; Pan et al., 2014); however, these sites may affect BAR activity.

Regulation of the floral scent synthesis pathway involves a complex network of enzymes, plant hormones, transcription factors and signal transduction proteins, among others. These elements work together to precisely regulate the synthesis of floral scent metabolites at the appropriate development stages and in specific tissues and organs of *P. mume*. Their differential expression can lead to differences in the floral scent metabolism network in different varieties of *P. mume*. As shown in Figure 8, the benzaldehyde content was all very high in the intracellular pools of the “Beijing Yudie” and “Xiangruibai” varieties, which exhibit the characteristic fragrance; in the “Dan Fenghou” and “Yanxing” varieties, which exhibit low fragrance; and in the “Fenghou” variety, which exhibits no fragrance. Notably, the direction and distribution of benzaldehyde metabolic flux determines the benzyl acetate content. Metabolic flux that favored the synthesis of benzyl benzoate led to decreases in benzyl acetate synthesis in the low-fragrance varieties “Dan Fenghou” and “Yanxing,” whereas low production of benzyl alcohol caused the lack of benzyl acetate synthesis in the non-fragrant variety “Fenghou.” The synthesis of eugenol, another component of the characteristic *P. mume* floral scent, and the synthesis of chavicol also formed metabolic-flux-like relationships. Compared with “Xiangruibai,” the metabolic flux in “Dan Fenghou” tended to favor the synthesis of chavicol, which caused the fragrance of this variety to differ from the characteristic floral scent of *P. mume*. 4CL3, CCR1, and HCT2 potentially involved in the synthesis of eugenol, while CCR3 might be involved in the synthesis of chavicol. Moreover, methyl chavicol and methyl salicylate were also detected among the volatiles of some varieties. These key steps in the floral scent synthesis pathway are regulated by some transcription factors (Zif, AP2/B3-like), stress-related factors (HSP18.2, RD22, SPDS, etc.), hormones (GA, ABA, auxin, BR, CK, Et), posttranslational modification enzymes (UBC32 and E3 ligases) and transporters (AMT1.2, PHT1.4, ZIP5, PDR3, ABC-2, BOR1, CNGC1.1, CNGC1.2, and MATE). The differential expression of the related genes likely caused the differences in floral scent among the different varieties of *P. mume*. Since a very limited number of genes related to benzenoids synthesis were identified in the correlation analysis between metabolites and transcriptome, we speculate that posttranslational modification may be an important strategy to regulate the synthesis network. It can directly affect the catalytic activity at the protein level, and then affect floral scent synthesis in *P. mume*. The mechanism how ubiquitin-mediated protein degradation regulates the floral scent metabolism in *P. mume* will be unfolded in our future work.

## Data Availability Statement

All raw-sequence read data were published as a BioProject (PRJNA610678) in the NCBI Sequence Read Archive under accession number SRP251790.

## Author Contributions

FB and QZ planned and designed the research. FB, TZ, AnD, and AiD performed the experiments. WY, JW, TC, and QZ supported the resources. FB wrote the manuscript. All authors contributed to the article and approved the submitted version.

## Conflict of Interest

The authors declare that the research was conducted in the absence of any commercial or financial relationships that could be construed as a potential conflict of interest.

## Footnotes

1 http://www.ncbi.nlm.nih.gov/Structure/cdd/wrpsb.cgi

2 http://mapman.gabipd.org/web/guest/mapcave

3 https://swissmodel.expasy.org/

4 http://www.rcsb.org/

## Supplementary Material

Supplementary Figure 1 The heat map of correlations between RNA-seq samples.

Supplementary Figure 2 GO significant enrichment analysis of the differentially expressed genes in the five varieties.

Supplementary Figure 3 KEGG pathway enrichment analysis of the differentially expressed genes in the five varieties.

Supplementary Figure 4 Comparison of *PmBAR1* coding sequences in the different varieties.

Supplementary Figure 5 Comparison of *PmBAR2* coding sequences in the different varieties.

Supplementary Figure 6 Comparison of *PmBAR3* coding sequences in the different varieties.

Supplementary Figure 7 Comparison of PhCCR1 and PmBAR amino acid sequences.

Supplementary Figure 8 qPCR analysis of genes highly associated with floral scent metabolites.

Supplementary Table 1 Headspace analysis of volatiles in five varieties of *P. mume* by HS-SPME-GC-MS.

Supplementary Table 2 Analysis of intracellular pools of floral scent metabolites in five varieties of *P. mume* by GC-MS.

Supplementary Table 3 Quality analysis of all reads from fifteen samples.

Supplementary Table 4 Numbers and percentages of the total clean reads mapped to the reference genome for fifteen samples.

Supplementary Table 5 Gene structure annotations of novel genes.

Supplementary Table 6 Novel isoforms of known genes.

Supplementary Table 7 Information on the key enzymes in the floral scent synthesis pathway.

Supplementary Table 8 Lists of the key enzyme-coding genes that were differentially expressed among the five varieties of *P. mume*.

Supplementary Table 9 Correlation matrix for metabolite levels and gene expression levels (*R*^2^ > 0.8, *P* < 0.01).

Supplementary Table 10 Interaction values between 6 metabolites and 100 selected genes.

Supplementary Table 11 Information on selected genes in the interaction networks.

Supplementary Table 12 The primer sequences for qPCR.

Supplementary Table 13 qPCR validation of transcript levels evaluated by RNA-seq.

## Abbreviations

4CL: 4-coumarate-CoA ligase
ABC-2: adenosine triphosphate-binding cassette transporter 2
ADH: benzyl alcohol dehydrogenase
AMT1.2: ammonium transporter 1.2
BAHD: beat, anthocyanin O-hydroxycinnamoyltransferase, anthocyanin O-hydroxyeinnamoyltransferase and deacetylvindoline-4-O-acetyltransferase
BAK1: BRI1-associated receptor kinase 1
BAR: benzaldehyde reductase
BEAT: benzyl alcohol acetyltransferase
BOR1: requires high boron 1
BPBT: benzyl alcohol/phenylethanol benzoyltransferase
CAD: coniferyl/caffeoyl/coumaryl-alcohol dehydrogenase
CCD7: carotenoid cleavage dioxygenase 7
CCR: cinnamoyl-CoA reductase
CFAT: coniferyl alcohol acetyltransferase
CKX3: cytokinin oxidase 3
CNGC1: cyclic nucleotide gated channel 1
CoA: coenzyme A
CYP72A: cytochrome P450 protein 72 A
DWF1: drawf 1
EGS: eugenol synthase
ERF: ethylene response factor
FPKM: fragments per kilo base per million base
GA3: GA-requiring 3
GA3OX: gibberellin 3-oxidase
HCT: hydroxycinnamoyl-CoA shikimate/quinate hydroxycinnamoyl transferase
HSP18.2: heat shock protein 18.2
MATE: MATE efflux
PAL: phenylalanine ammonia-lyase
PAR: phenylacetaldehyde reductase
PCA: principal component analysis
PDR3: pleiotropic drug resistance protein 3
PHT1.4: phosphate transporter 1.4
PP2C: protein phosphatase 2C
qPCR: quantitative PCR
RD22: responsive to dehydration 22
SAMT1: salicylic acid carboxyl methyltransferase 1
SDR: short-chain reductase
SERK2: somatic embryogenesis receptor-like kinase 2
SPDS: spermidine synthase
SUMO2: small ubiquitin-like modifier 2
UBC32: ubiquitin-conjugating enzyme 32
UGT: UDP-glucosyl transferase
Zif: zinc finger type transcription factor
ZIP5: zinc transporter 5.
