# Supplementary material for: Metabolic, Enzymatic Activity, and Transcriptomic Analysis Reveals the Mechanism Underlying the Lack of Characteristic Floral Scent in Apricot Mei Varieties
Source: Front Plant Sci. 2020 Oct 22;11:574982. doi: 10.3389/fpls.2020.574982 (PMC7642261; doi:10.3389/fpls.2020.574982)
Supplement: Supplementary Figure 1 — The heat map of correlations between RNA-seq samples. [file Data_Sheet_1.docx]

**
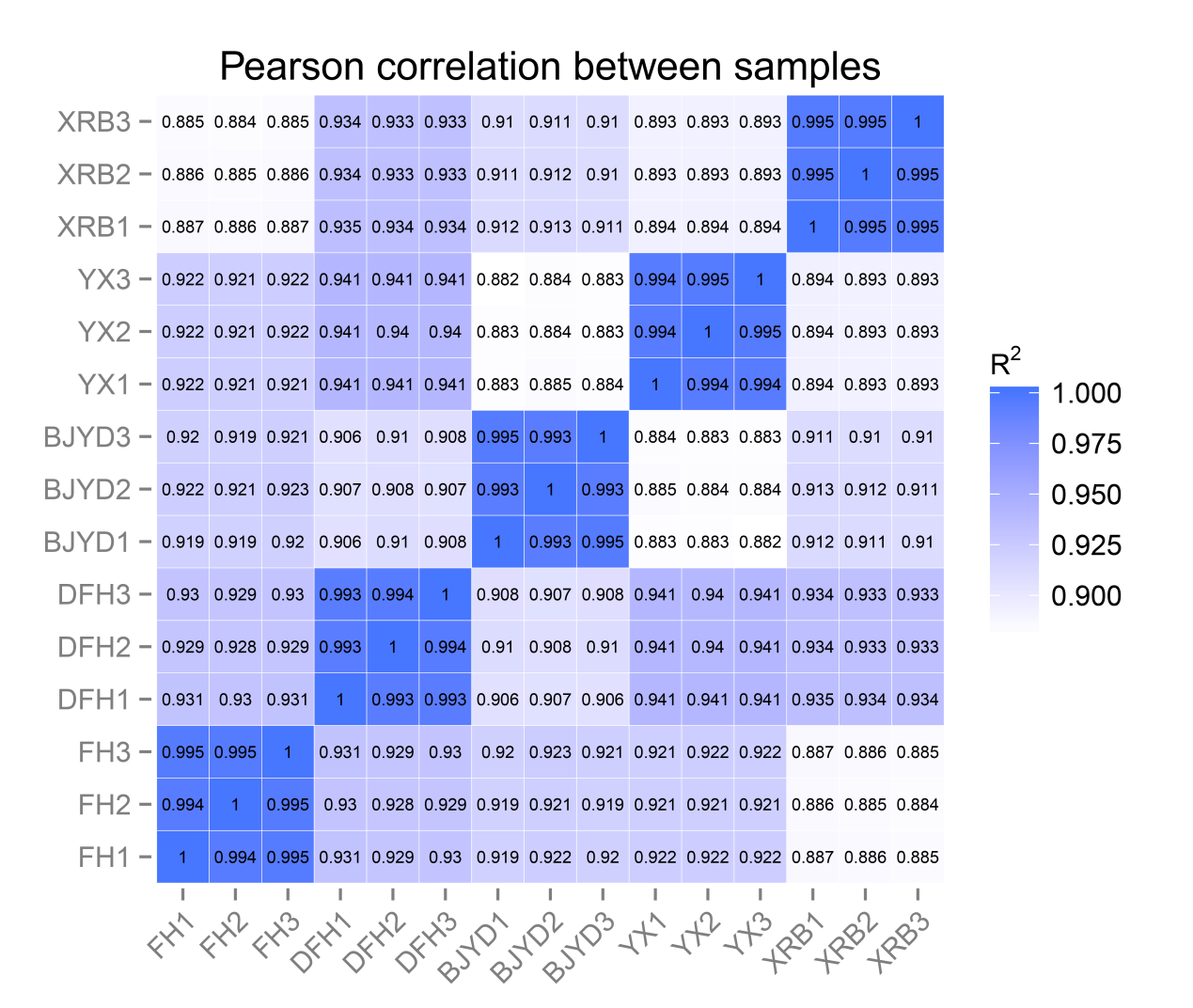
**

**Figure S1.** **The heat map of correlations between RNA-seq samples.**

The squares of Pearson correlation coefficients between each two samples were showed in the array.

**
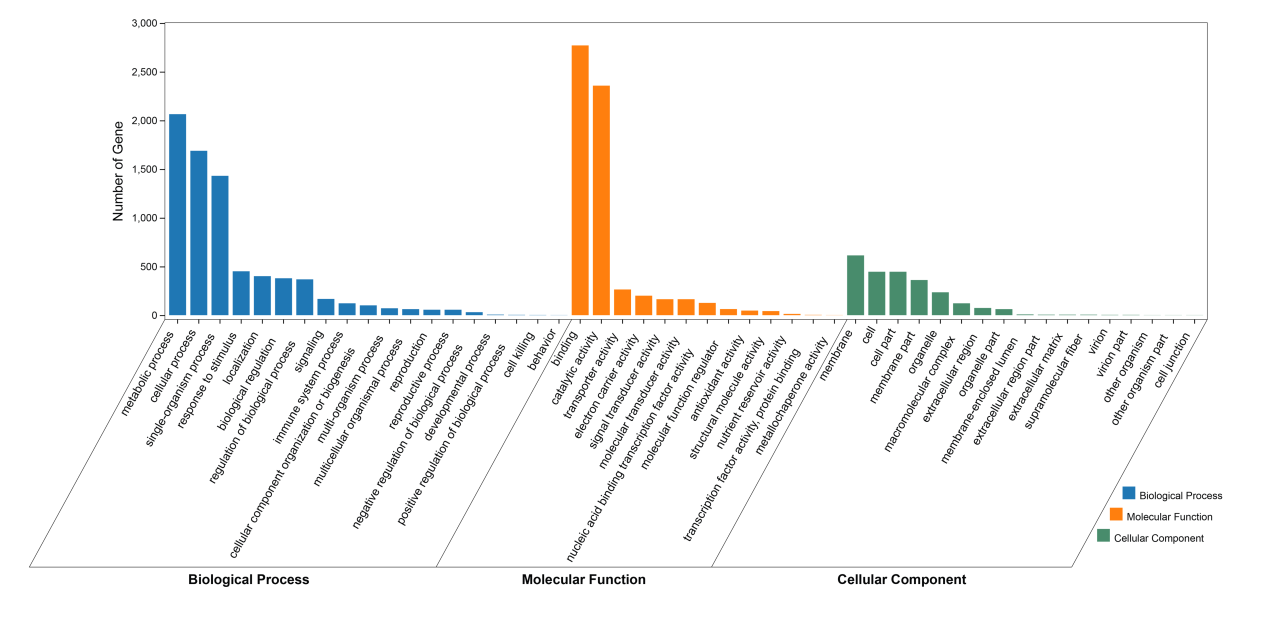
**

**Figure S2. GO significant enrichment analysis of the differentially expressed genes in the five varieties.**

GO contents three term types: Biological process (blue bar), Molecular function (orange bar) and Cellular component (green bar). Adjusted P-Value < 0.05.**
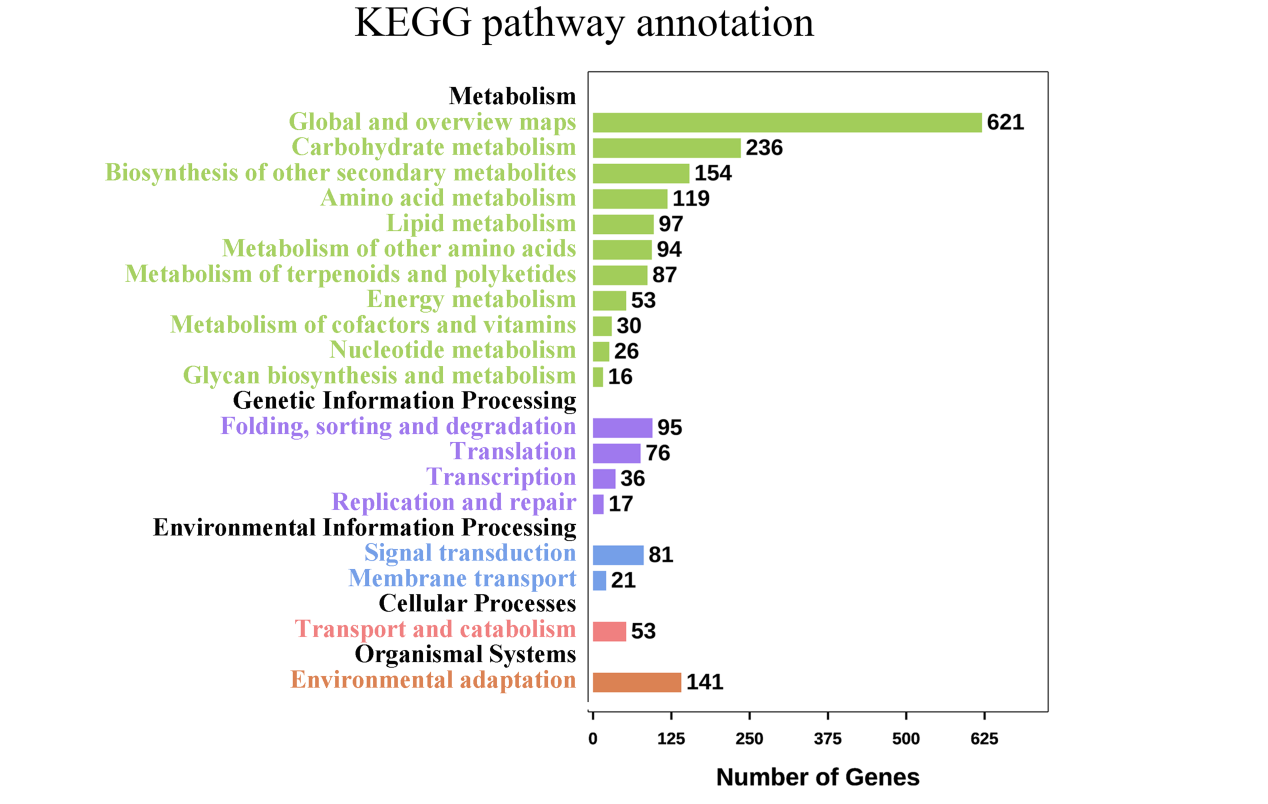
**

**Figure S3. KEGG pathway enrichment analysis of the differentially expressed genes in the five varieties.**

Five types of KEGG pathway are showed, including Metabolism (green bar), Genetic information processing (purple bar), Environmental information processing (blue bars), Cellular processes (pink bar) and Organismal systems (brown bar). The number of gene in each category is indicated beside the bars.


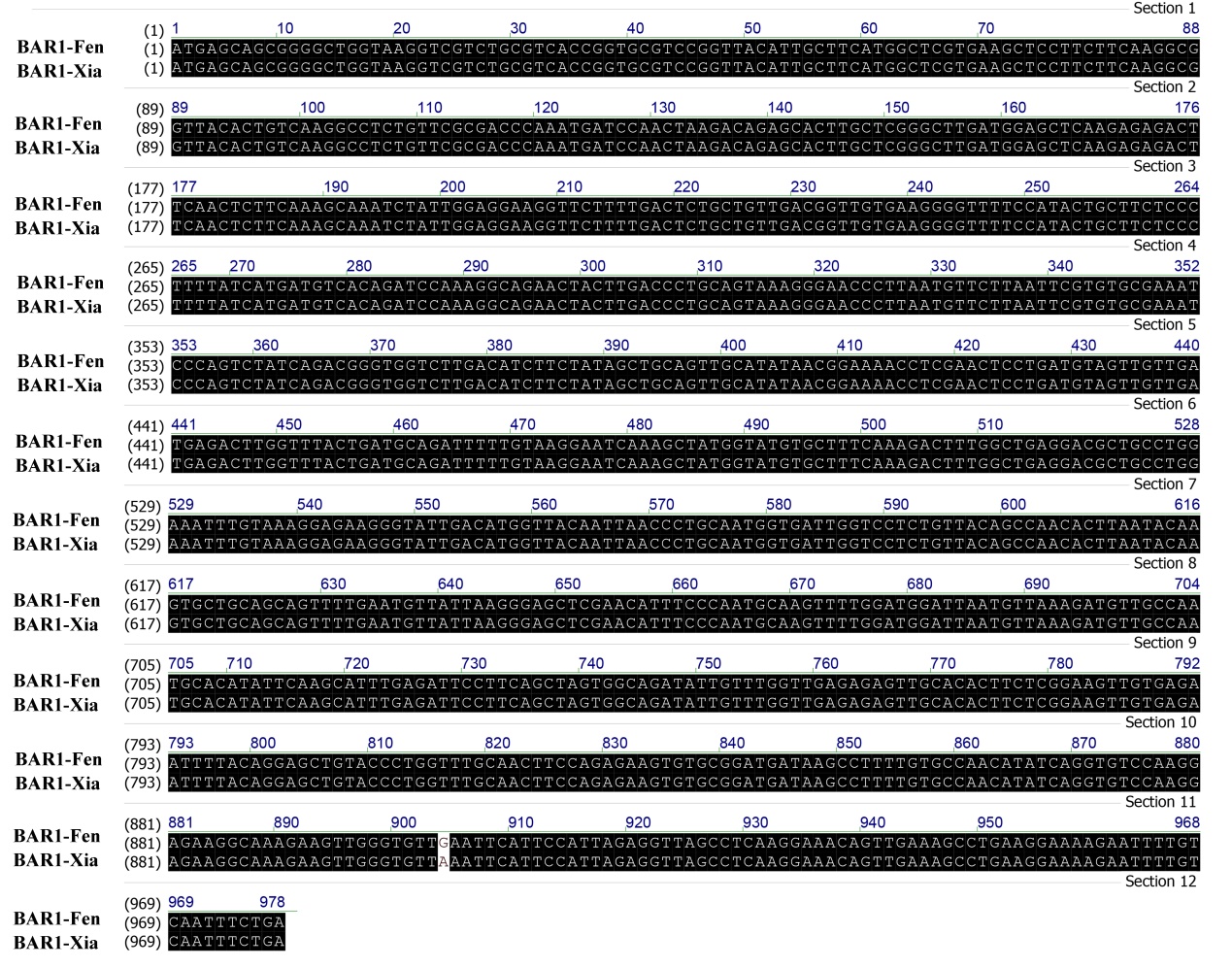


**Figure S4. Comparison of *PmBAR1* coding sequences in the different varieties.**

The conservative residues are showed in black background. The non-similar residues are showed in white background.


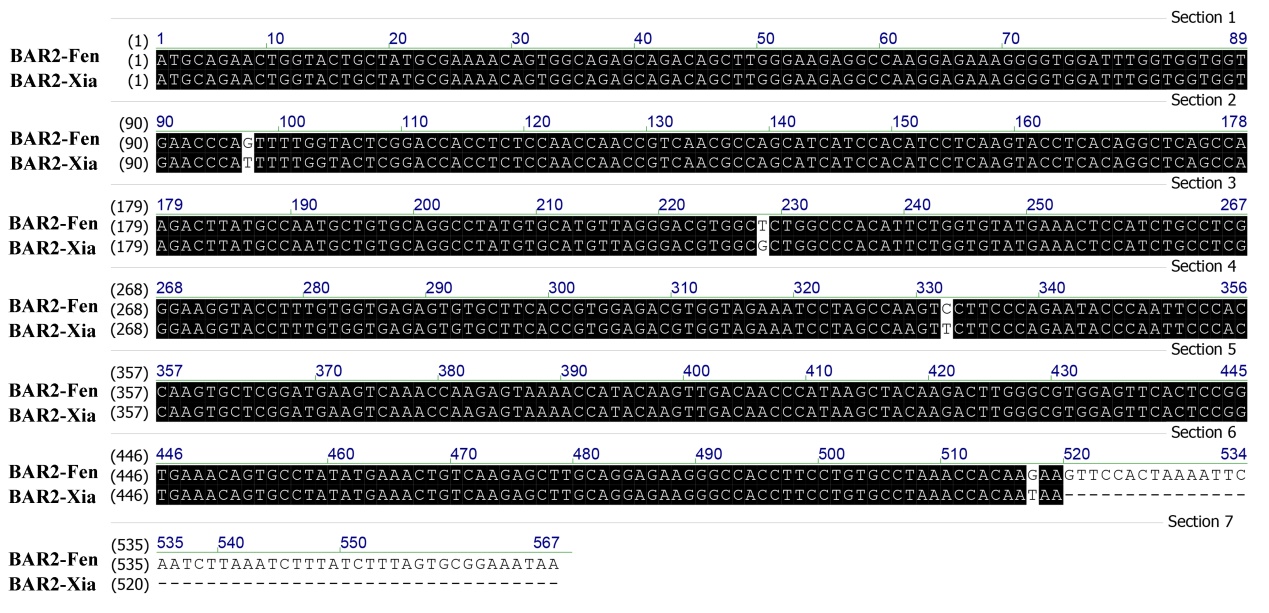


**Figure S5. Comparison of *PmBAR2* coding sequences in the different varieties.**

The conservative residues are showed in black background. The non-similar residues are showed in white background.


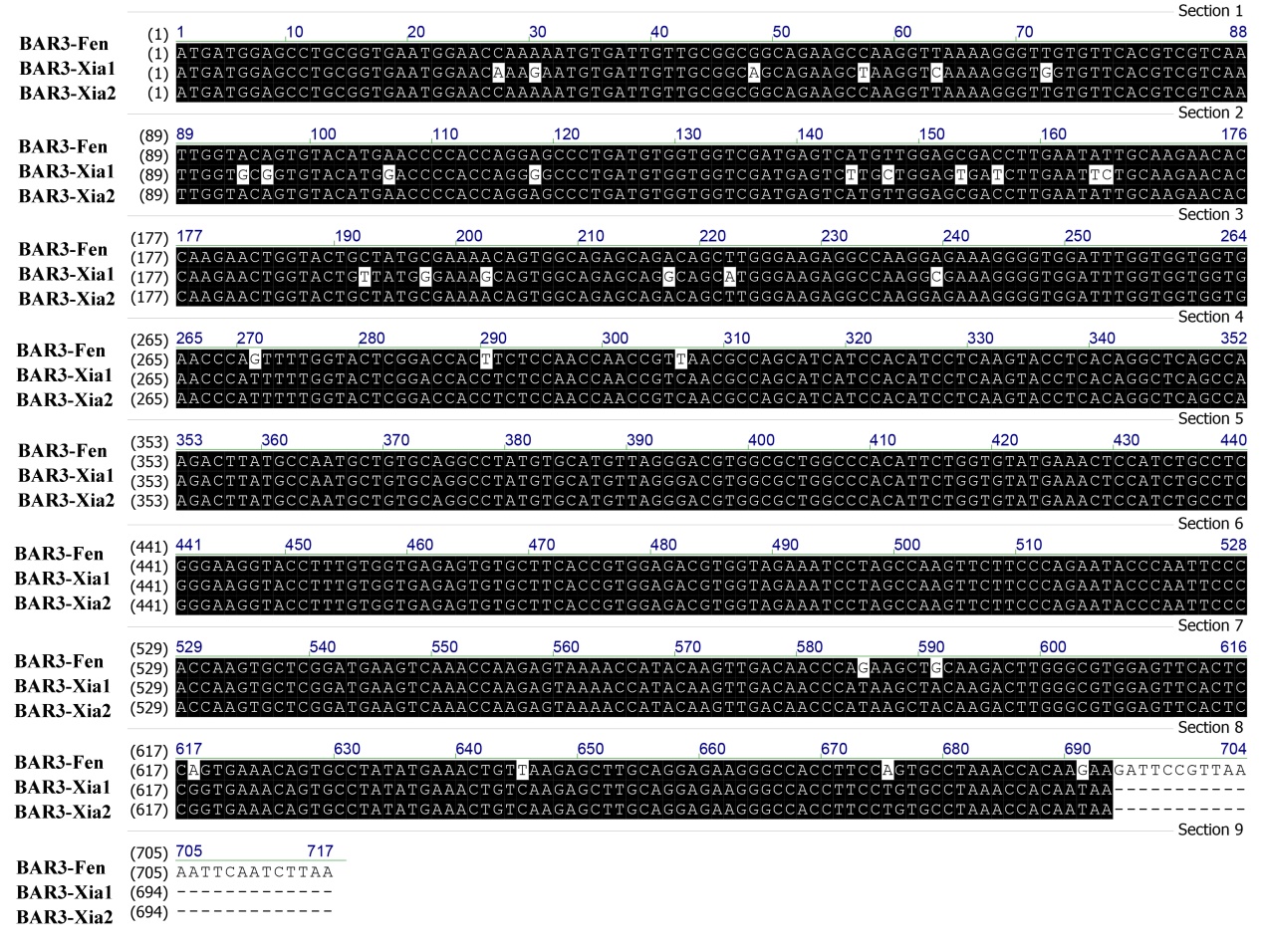


**Figure S6. Comparison of *PmBAR3* coding sequences in the different varieties.**

The conservative residues are showed in black background. The non-similar residues are showed in white background.


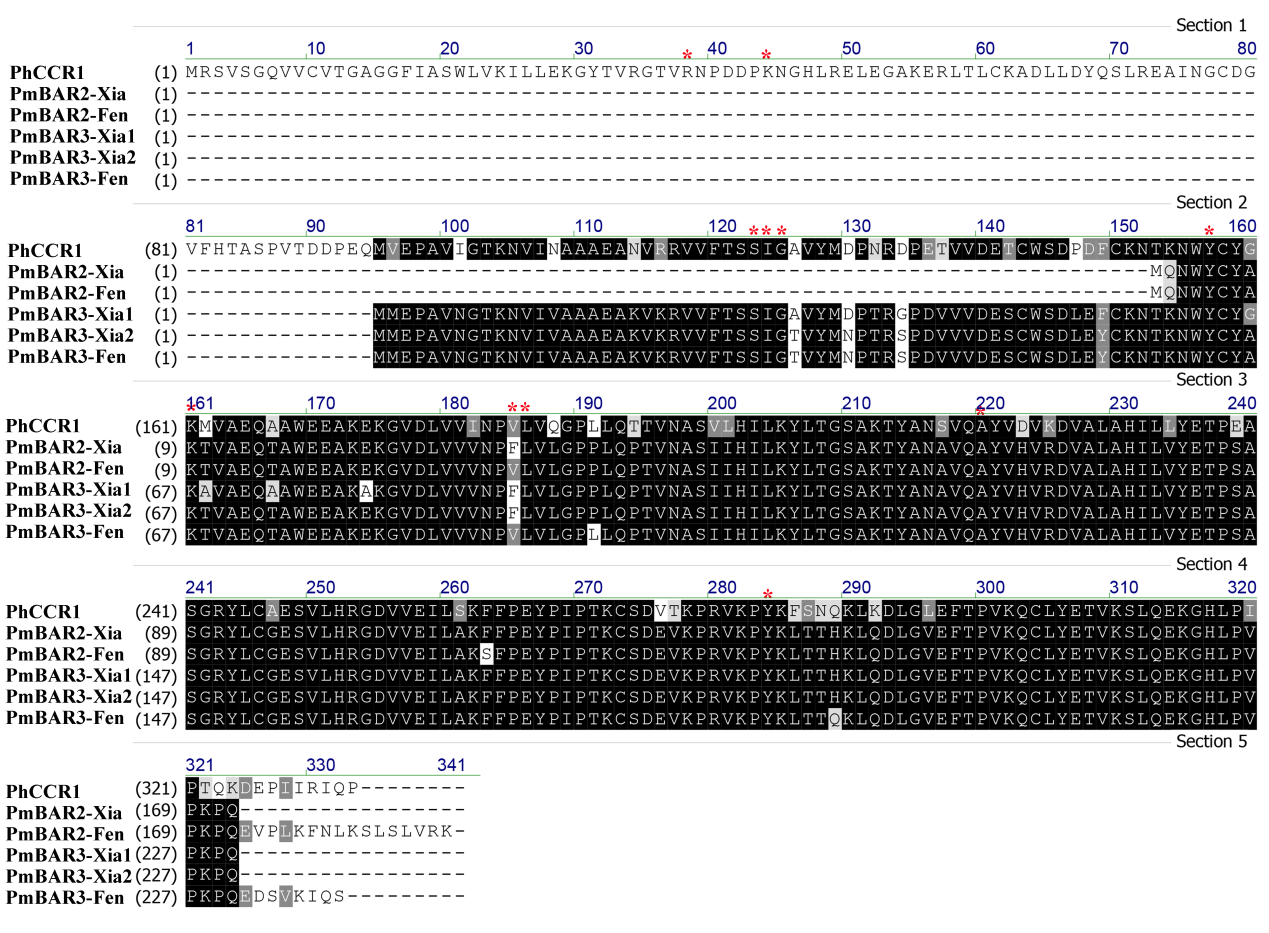


**Figure S7. Comparison of PhCCR1 and PmBAR amino acid sequences.**

The conservative residues are showed in black background. The similar residues are showed in gray background. The non-similar residues are showed in white background. The red stars indicate reported sites that can affect substrate recognition and CCR catalytic activity.

**
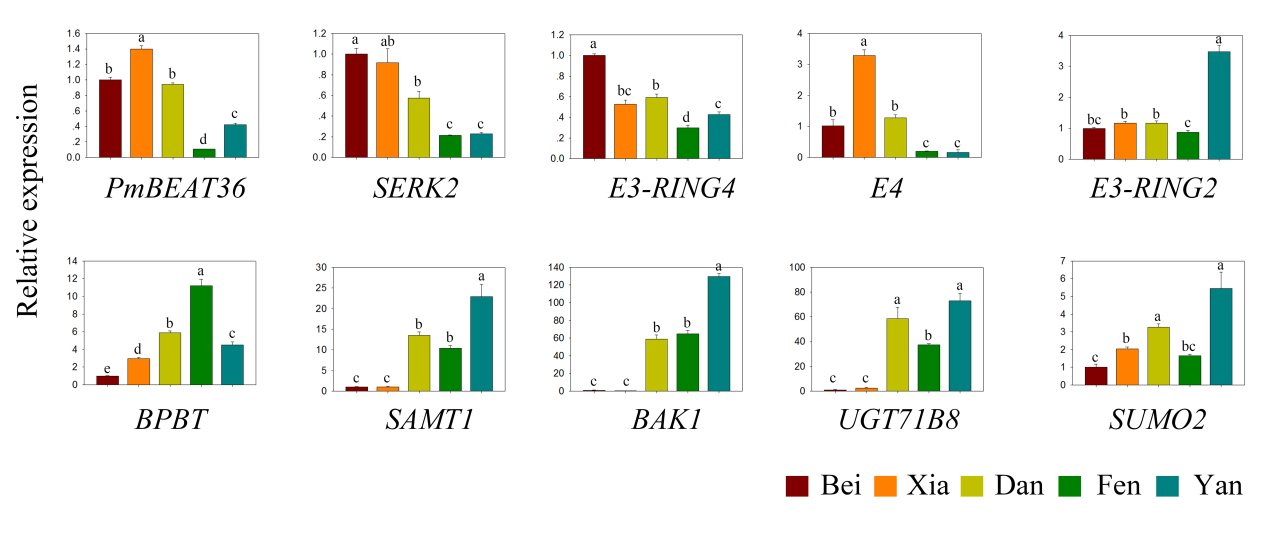
**

**Figure S8. qPCR analysis of genes highly associated with floral scent metabolites.**

The gene *PmPP2A* was used as a reference gene. The data are presented as the mean values of three replicates ± SD. Three independent experiments were performed with similar results. Different letters indicate significant differences at the P=0.05 level.
